# Aberrant Functional Connectivity of the Posterior Cingulate Cortex in Type 2 Diabetes Without Cognitive Impairment and Microvascular Complications

**DOI:** 10.3389/fendo.2021.722861

**Published:** 2021-10-25

**Authors:** Panpan Cheng, Shuyan Song, Yumin Li, Yao Zhang, Jun Yi, Xiangyang Xu, Hongmei Zhou, Zhentao Zuo

**Affiliations:** ^1^ Department of Radiology, Liyuan Hospital, Tongji Medical College, Huazhong University of Science and Technology, Wuhan, China; ^2^ College of Electrics and Information Engineering, South-Central University for Nationalities, Wuhan, China; ^3^ Department of Radiology, Union Hospital, Tongji Medical College, Huazhong University of Science and Technology, Wuhan, China; ^4^ Department of Endocrinology, Liyuan Hospital, Tongji Medical College, Huazhong University of Science and Technology, Wuhan, China; ^5^ Department of Psychiatry, Liyuan Hospital, Tongji Medical College, Huazhong University of Science and Technology, Wuhan, China; ^6^ State Key Laboratory of Brain and Cognitive Science, Beijing MRI Center for Brain Research, Institute of Biophysics, Chinese Academy of Sciences, Beijing, China; ^7^ Sino-Danish College, University of Chinese Academy of Sciences, Beijing, China; ^8^ CAS Center for Excellence in Brain and Science and Intelligence Technology, Chinese Academy of Sciences, Beijing, China

**Keywords:** type 2 diabetes mellitus, posterior cingulum cortex, resting-state functional MRI, functional connectivity, cognitive impairment

## Abstract

**Objective:**

We aimed to investigate the alterations of brain functional connectivity (FC) in type 2 diabetes mellitus (T2DM) patients without clinical evidence of cognitive impairment and microvascular complications (woCIMC-T2DM) using resting-state functional MRI (rs-fMRI) and to determine whether its value was correlated with clinical indicators.

**Methods:**

A total of 27 T2DM and 26 healthy controls (HCs) were prospectively examined. Cognitive impairment was excluded using the Mini-Mental State Examination (MMSE) and the Montreal Cognitive Assessment (MoCA) scales, and microvascular complications were excluded by fundus photography, microalbuminuria, and other indicators. The correlation maps, derived from rs-fMRI with posterior cingulate cortex (PCC) as the seed, were compared between T2DM patients and HCs. Pearson’s correlation analysis was performed to determine the relationship between the FC of PCC and the clinical indicators.

**Results:**

Compared with HC, woCIMC-T2DM patients showed significantly decreased FCs with PCC (PCC-FCs) in the anterior cingulate cortex (ACC), right superior frontal gyrus, right medial frontal gyrus, and right angular gyrus. Meanwhile, increased PCC-FCs was observed in the right superior temporal gyrus and calcarine fissure (CAL). The FC of PCC-ACC was negatively correlated with glycosylated hemoglobin (HbA1c) and diabetes duration, and the FC of PCC-CAL was significantly positively correlated with HbA1c and diabetes duration.

**Conclusion:**

The FC, especially of the PCC with cognitive and visual brain regions, was altered before clinically measurable cognitive impairment and microvascular complications occurred in T2DM patients. In addition, the FC of the PCC with cognitive and visual brain regions was correlated with HbA1c and diabetes duration. This indicates that clinicians should pay attention not only to blood glucose control but also to brain function changes before the occurrence of adverse complications, which is of great significance for the prevention of cognitive dysfunction and visual impairment.

## 1 Introduction

According to the prediction of the International Diabetes Association, the total number of diabetes patients worldwide will reach 642 million by 2040, of which type 2 diabetes mellitus (T2DM) accounts for 90%–95% (1). T2DM is a chronic metabolic disorder characterized by hyperglycemia, which can lead to complications associated with the peripheral nerves and blood vessels, even affecting the brain structure and function (2). Brain morphological studies based on magnetic resonance imaging (MRI) have shown a reduction in gray matter density (3) and volume (4), a thinning of cortical thickness (5), and abnormal microstructure of the white matter (6) in T2DM patients compared with healthy controls (HC). However, these brain damages are not unique to diabetes, but also occur in other diseases such as psychiatric disorders and mild cognitive impairment (MCI) (7). Resting-state functional MRI (rs-fMRI) makes up for the deficiency of structural MRI, to some extent.

rs-fMRI is a promising and noninvasive neuroimaging technique for evaluating spontaneous neural activity through blood oxygen level-dependent (BOLD) contrast during resting state (8). Independent component analysis found disrupted functional connectivity (FC) in T2DM across multiple networks, involving the salience network, attentional control network, left frontal–parietal network, sensorimotor network, and the default mode network (DMN) (9). The DMN, including the posterior cingulate cortex (PCC), the precuneus, ventral medial prefrontal cortex, and the inferior parietal lobe (10), is a widely studied network that is activated during the resting state and is related to several psychological functions such as attention and cognition (11). Previous studies (9, 12) showed that the functional activity and the connectivity within the DMN in T2DM patients were abnormal compared with those in HCs.

T2DM is often accompanied by MCI (13) and microvascular complications (14). However, MCI, which are not induced by T2DM, can also lead to brain function changes in the DMN (7). It is still unclear whether diabetes-related abnormal brain function is caused by concomitant MCI and microvascular complications or by the metabolism of diabetes itself. In addition, most studies predominantly focused on the aberrant brain changes in T2DM patients concerning cognitive impairment (12, 15). Recently, a few studies (14, 16) have been performed to explore the effects of T2DM without either cognitive impairment or microvascular complications. Network-based analysis showed that the decreased connectivity strength within the DMN was found exclusively between HCs and T2DM patients with MCI (16). However, the connectivity strength within the DMN was not significantly reduced in T2DM patients without MCI. It seems that the reduced functional synchronization in T2DM patients is caused by cognitive impairment.

PCC-based FC (PCC-FC) analysis, as a widely employed method to define the DMN, is increasingly used to explore the abnormal whole brain FC connected with PCC in clinical studies (12, 15). To our knowledge, there is yet no study investigating the aberrant function synchronization of T2DM patients without clinical evidence of cognitive impairment and microvascular complications (woCIMC-T2DM) using PCC-FC. In this study, T2DM patients without cognitive impairment and microvascular complications were enrolled. Functional synchronization was compared between woCIMC-T2DM patients and HCs with PCC-FC, and aberrant local PCC-FC was used to predict the clinical indicator through correlation.

## 2 Methods

### 2.1 Subjects

This study was approved by the Research Ethics Committee of the Affiliated Liyuan Hospital, Tongji Medical College, Huazhong University of Science and Technology. All subjects provided written informed consent before their participation in the study.

**Figure 1:**
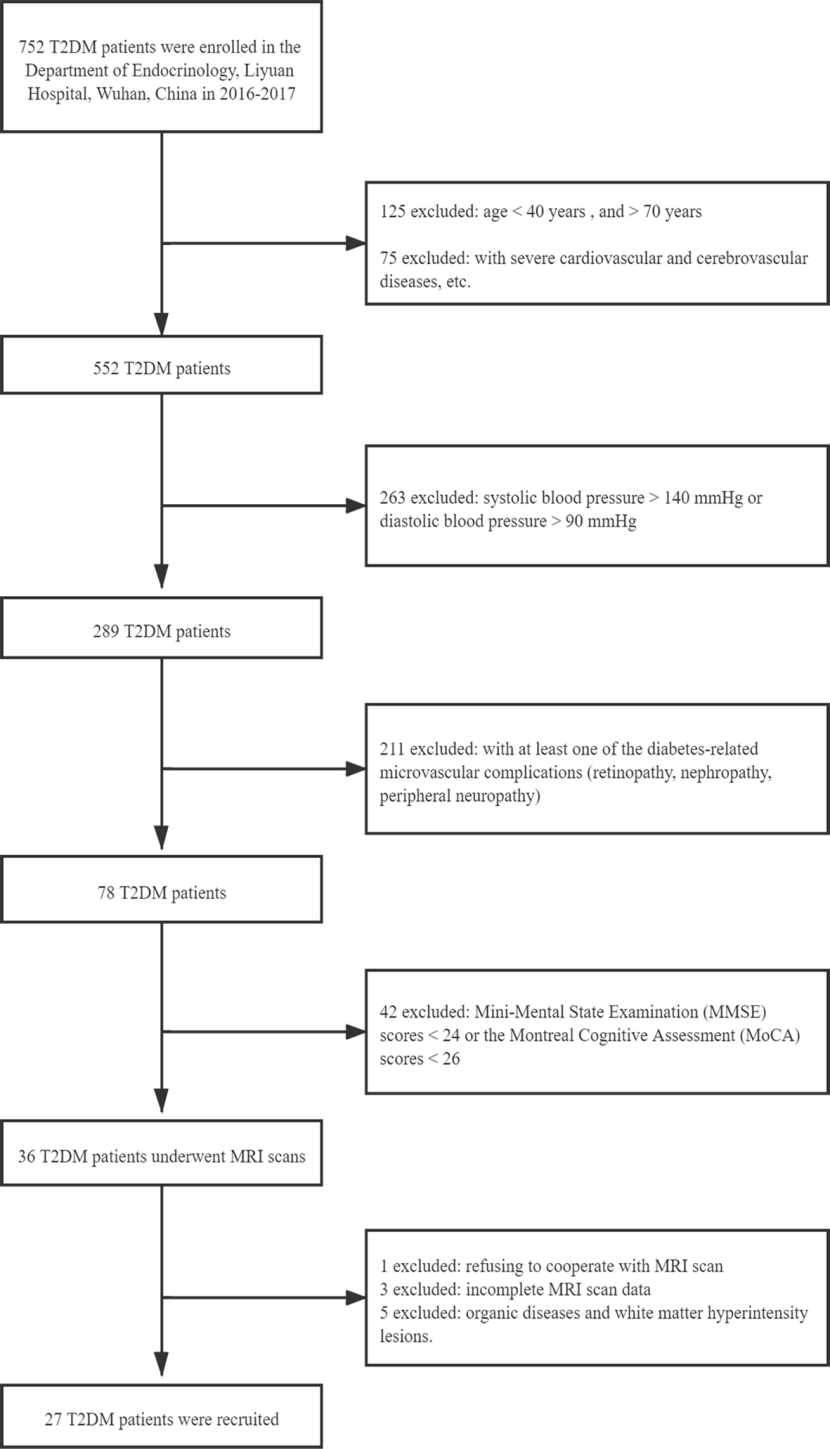
Flowchart of the exclusion and inclusion criteria for type 2 diabetes mellitus (T2DM) patients without clinical evidence of cognitive impairment and microvascular complications.

A total of 53 subjects were recruited from the Department of Radiology, Liyuan Hospital, Tongji Medical College, Huazhong University of Science and Technology, Wuhan, China, from June 2016 to June 2017. In this cross-sectional study, all participants were enrolled according to the following criteria: 1) aged between 40 and 75 years, no less than 6 years of education to ensure the literacy of all subjects; 2) Mini-Mental State Examination (MMSE) score ≥24 and Montreal Cognitive Assessment (MoCA) score ≥26 (both scales are Chinese versions) (17, 18); 3) no history of psychiatric diseases, stroke, epilepsy, brain trauma and surgery, cerebrovascular accidents, or signs of impairment of cognitive function, or severe liver, kidney, or heart disease, or severe hyperglycemia coma and hypoglycemia; 4) no history of hypertension or hypertension medication: systolic blood pressure <140 mmHg and diastolic blood pressure <90 mmHg; 5) ability to meet the physical demands of the imaging procedure; and 6) walking independently, right-handedness, and non-blindness. Type 2 diabetes was diagnosed based on the criteria announced by the WHO 1999 (19). Fifty-three participants met these criteria, including 27 woCIMC-T2DM patients (14 females, age = 60.30 ± 8.26 years, range = 43–73 years) (**Figure 1**) and 26 age- and gender-matched healthy control participants with (13 females, age = 58.69 ± 9.55 years, range = 40–72 years).

### 2.2 Medical History and Biometric Measurements

All participants underwent a medical history and physical examination, including height, weight, and body mass index (BMI). Blood pressure was measured three times on different days with systolic blood pressure <140 mmHg and diastolic blood pressure <90 mmHg. Biometric measurements, such as averaged fasting blood glucose (FBG), glycosylated hemoglobin (HbA1c), total cholesterol, triglycerides, high-density lipoprotein, and low-density lipoprotein, were measured using standard laboratory testing.

### 2.3 Cognitive Assessment

In this study, the MMSE was used to screen dementia (17) and the MoCA used to evaluate MCI (18). MMSE mainly includes the following seven aspects: time orientation, location orientation, immediate memory, attention and calculation, delayed memory, language, and visual space. All subjects with scores <24 were excluded. MoCA comprised the following eight cognition domains: visual space, executive function, naming, attention, language, abstraction, delayed memory, and orientation. If a subject had less than 12 years of education, 1 point was added to the total score. Those with total score <26 points were excluded. The cognitive scales were assessed by one experienced psychiatrist (JY).

### 2.4 MRI Data Acquisition

The MR imaging dataset was acquired with a 3.0-T MRI scanner (Verio, Siemens Healthineers, Erlangen, Germany) with a commercial 12-channel head array coil. All of the subjects were instructed to relax their minds and keep their eyes closed while awake during scanning. Pairs of foam padding and earplugs were used to reduce head motion and scanner noise, respectively. The brain sagittal T1-weighted, axial T1-weighted, T2-weighted, and T2-weighted fluid-attenuated inversion recover (T2-FLAIR) images were scanned for every subject in order to exclude organic diseases and white matter hyperintensity (WMH) lesions. Functional images were obtained using a gradient-echo planar imaging sequence, parallel to the AC–PC (anterior commissure–posterior commissure), with the following imaging parameters: repetition time (TR) = 2,000 ms, echo time (TE) = 30 ms, slices = 32, thickness = 3 mm, gap = 0.9 mm, field of view (FOV) = 200 mm × 200 mm, matrix = 64 × 64, and flip angle (FA) = 90°C, with scan time of 8 min and 6 s and 240 volumes for each subject. The parameters for T1-weighted three-dimensional spoiled gradient-echo sequences were as follows: TR = 2,300 ms, TE = 2.27 ms, slices = 192, thickness = 1 mm, gap = 0.5 mm, FOV = 250 mm × 250 mm, matrix = 256 × 256, and FA = 8°C. T2-FLAIR scanning parameters were: TR = 8,000 ms, TE = 95 ms, inversion time (TI) = 2,500 ms, slices = 19, thickness = 5 mm, FOV = 230 mm × 230 mm, matrix = 192 × 192, and FA = 150°C.

### 2.5 Microvascular Complication Assessment

Diabetic retinopathy was diagnosed by fundus photography and graded according to the Wisconsin Epidemiologic Study classification (20). Only those patients with a score of 0 (no retinopathy) were included in this study. Diabetic nephropathy was assessed with microalbuminuria, which was defined by an albumin-to-creatinine ratio >30 mg/g. Diabetic peripheral neuropathy was diagnosed as a score ≥6 on the Toronto Clinical Neuropathy Scoring System (21). Subjects were graded according to neuropathy severity using six symptom scores (the presence or absence of foot pain, numbness, tingling, weakness, imbalance, and upper limb symptoms), eight reflex scores (bilateral knee and ankle reflexes, each graded as absent, reduced, or normal), and five physical examination scores (the presence or absence of pinprick, temperature, light touch, vibration, and position sense), for a total of 19 possible points. Only patients with a score <6 (no peripheral neuropathy) were included in this study.

No clinical evidence of retinopathy, nephropathy, and peripheral neuropathy was detected in all subjects.

### 2.6 Functional Data Analysis

Functional data were analyzed using DPARSF_V2.0 (22) (http://www.restfmri.net/forum/DPARSF), REST_V1.6 (http://www.restfmri.net) and SPM (http://www.fil.ion.ucl.ac.uk/spm) on MATLAB R2012a (MathWorks, Natick, MA, USA).

The main preprocessing included: 1) slice timing after removing the first 10 time points; 2) head motion correction using realignment (average head motions >3.0 mm in translation or 3.0°C in rotation were excluded); 3) spatial normalization to the Montreal Neurological Institute (MNI) space and resampling the voxel size to 3 mm isotropic; 4) spatial smoothing with Gaussian kernel of 4 mm; 5) linear detrending and band-pass filtering (0.01–0.08 Hz); and 6) regressing out nuisance covariates (including six head motions, global mean signal, white matter signal, and cerebrospinal fluid signal).

**Figure 2:**
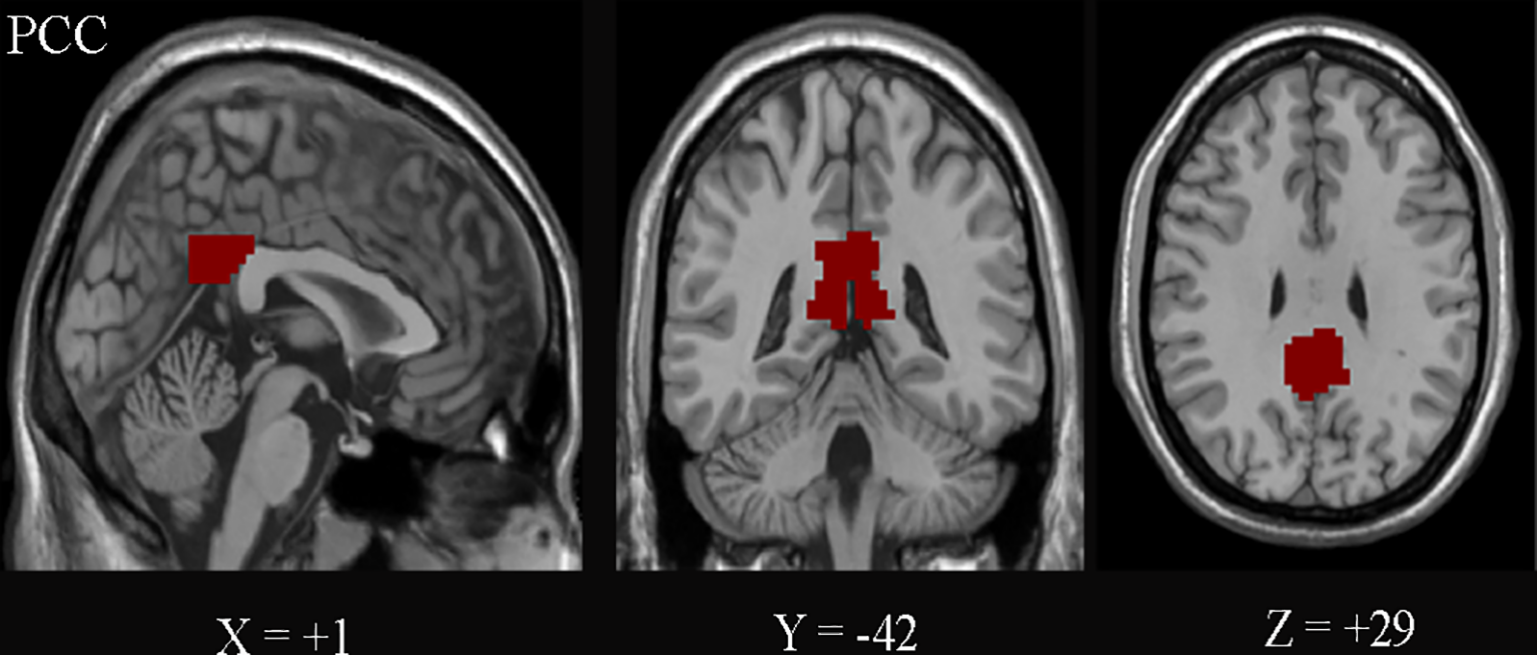
The posterior cingulate cortex (PCC) on the Montreal Neurological Institute (MNI) coordinates (+1, −42, and +29) is shown.

The PCC was defined as the seed regions of interest (ROIs) using the WFU_PickAtlas software (http://www.ansir.wfubmc.edu) in MNI standard space (**Figure 2**). PCC was also used as the seed in previous studies (12, 15, 23). The averaged time series of PCC was extracted as the reference. Pearson’s correlation coefficients were calculated between the mean signal of PCC and the time series of the whole brain voxels. Finally, Fisher’s *z*-transformation on the correlation coefficients was performed to improve the normality for statistics.

### 2.7 Statistical Analysis

#### 2.7.1 Demographic and Clinical Characteristics Analysis

The demographic and clinical variables were compared between T2DM patients and HCs using SPSS v24.0 (IBM Inc., Chicago, IL, USA). Continuous variables were tested using independent samples *t*-test, while sex differences were examined using the chi-square test (significance was set at *p* < 0.05).

#### 2.7.2 Functional Connectivity Analysis

For intra-group analysis, one-sample *t*-test was carried out on the individual *z*-values using REST_V1.6 software to determine the brain regions showing significant FC with PCC. For inter-group analysis, the two-sample *t*-test was performed on the *z*-values to identify the regions with significantly different PCC-FC between T2DM patients and HCs (age, sex, education levels, and BMI were included as covariates). The demographics and the clinical data of the two groups were in accord with normal distribution. Thresholds were set to *p* < 0.05, with AlphaSim correction (minimum cluster size of 85 voxels).

#### 2.7.3 Correlation Analysis

ROI-based correlation analysis was used to investigate the relationship between the clinical indicators and PCC-FC of the above brain regions. The average *z*-values of PCC-FC in abnormal regions were calculated individually for T2DM. Pearson’s correlation coefficients between the PCC-FC and diabetic duration, FBG, and HbA1c were analyzed using SPSS. *P* < 0.05 was considered statistically significant.

## 3 Results

### 3.1 Demographic and Clinical Characteristics

**Table 1** summarizes the demographic and clinical characteristics of T2DM patients and HCs. No significant differences were identified in terms of age, sex, education level, BMI, blood pressure, total cholesterol (TC), triglyceride (TG), high-density lipoprotein (HDL), low-density lipoprotein (LDL), cerebrovascular disease, and cognitive functions between T2DM patients and HCs, while FBG (11.43 ± 2.95 mmol/L) and HbA1c (8.74 ± 2.35%) in T2DM patients were significantly (*p* < 0.001 and *p* < 0.001, respectively) higher than those in HCs (3.83 ± 0.61 and 4.36 ± 0.29, respectively).

**Table 1:** Summary of the demographic and clinical data.

	Type 2 diabetes (*n* = 27)	Healthy controls (*n* = 26)	*t*/*χ* ^2^ values	*p*-values
Age (years)	60.30 ± 8.26	58.69 ± 9.55	0.84	0.36
Sex (male/female)	13/14	13/13	0.02^a^	0.89
Height (cm)	164.26 ± 5.95	166.23 ± 6.67	1.16	0.29
Weight (kg)	58.78 ± 7.76	57.38 ± 7.14	0.37	0.54
BMI (kg/m^2^)	21.70 ± 1.77	20.72 ± 1.94	0.32	0.57
Education level (years)	12.37 ± 3.20	12.38 ± 3.18	0.01	0.93
Systolic blood pressure (mmHg)	130.22 ± 5.60	128.31 ± 6.30	0.28	0.60
Diastolic blood pressure (mmHg)	76.81 ± 6.43	77.69 ± 6.96	0.04	0.85
Diabetes duration (years)	7.59 ± 5.10	–	–	–
FBG (mmol/L)	11.43 ± 2.95	3.83 ± 0.61	85.27	<0.001*
HbA1c (%)	8.74 ± 2.35	4.36 ± 0.29	54.70	<0.001*
TC (mmol/L)	4.28 ± 0.81	4.31 ± 0.99	2.10	0.15
TG (mmol/L)	1.37 ± 0.61	1.35 ± 0.49	0.98	0.33
HDL cholesterol (mmol/L)	1.35 ± 0.23	1.28 ± 0.41	2.23	0.14
LDL cholesterol (mmol/L)	2.69 ± 0.81	2.24 ± 0.82	0.60	0.44
MMSE	28.86 ± 0.80	29.43 ± 0.73	0.03	0.86
MOCA	27.36 ± 1.01	28.23 ± 1.14	0.15	0.70

Data are represented as the mean ± SD or n (%).

BMI, body mass index; FBG, fasting blood glucose; HbA1c, glycosylated hemoglobin; TC, total cholesterol; TG, triglyceride; HDL, high-density lipoprotein; LDL, low-density lipoprotein; MMSЕ, Mini-Mental State Examination; MоСA, Montreal Cognitive Assessment.

*p < 0.05.

a Chi-square test.

### 3.2 Functional Connectivity Based on PCC

**Figure 3:**
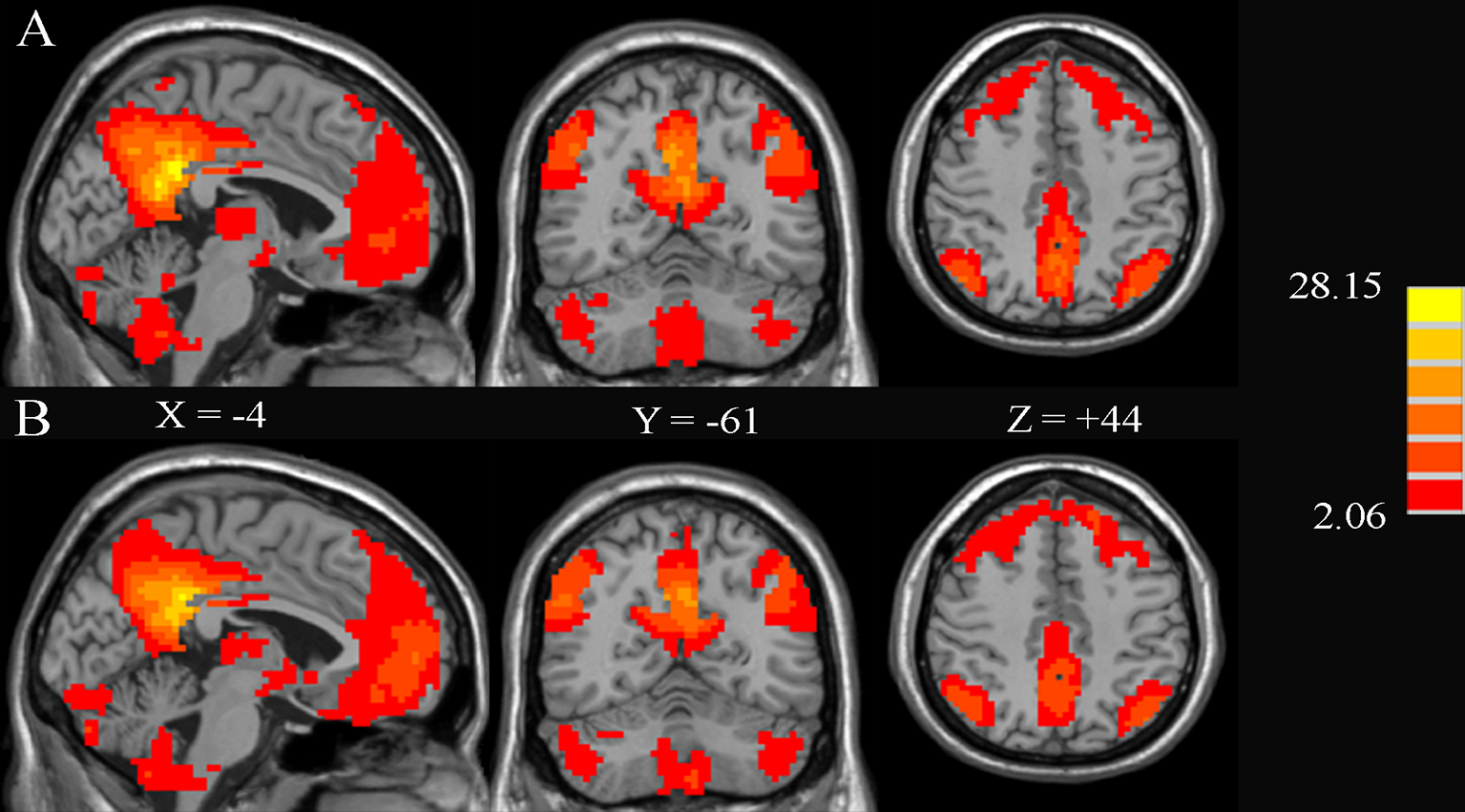
Significant brain functional connectivity with the posterior cingulate cortex (PCC) using one-sample *t*-test in healthy controls **(A)** and type 2 diabetes patients without cognitive and microvascular complications **(B)**. AlphaSim-corrected threshold of *p* < 0.05. The values of *X*, *Y*, and *Z* represent the MNI coordinates of the sagittal, coronal, and axial positions, respectively.

**Figure 3** shows the significant FC maps with PCC for T2DM patients and HCs. These regions included PCC, the anterior cingulate cortex (ACC), precuneus, ventral medial prefrontal lobe, bilateral inferior parietal lobe, and angular gyrus, which were in line with the DMN regions (10). Besides, several other regions, such as the cerebellum posterior lobe and bilateral calcarine fissure (CAL), were also detected.

**Figure 4:**
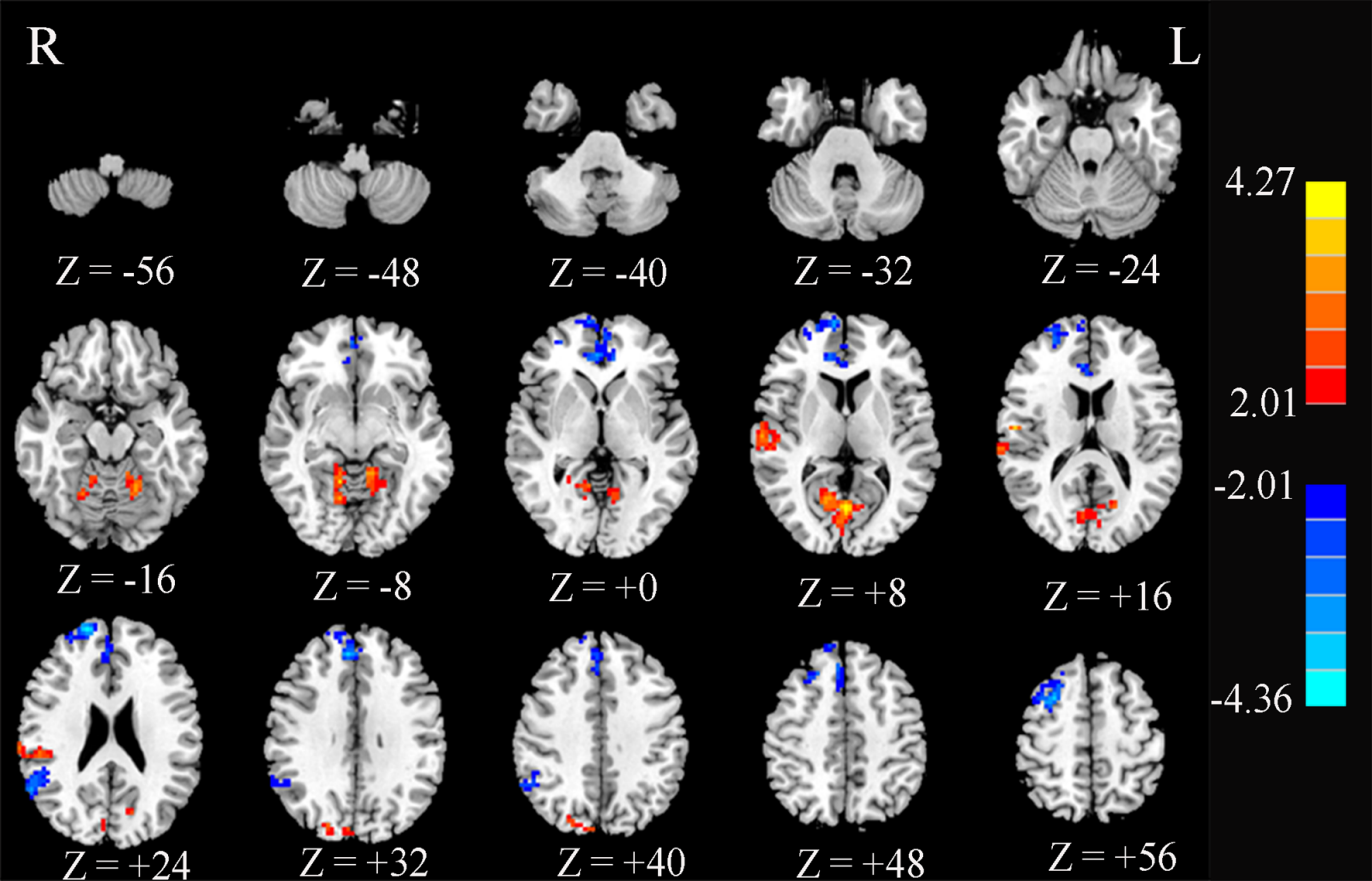
Significant differences in the functional connectivity of the posterior cingulate cortex (PCC) between type 2 diabetes patients without cognitive and microvascular complications and healthy controls. AlphaSim-corrected threshold of *p* < 0.05. *Z* represents the Montreal Neurological Institute (MNI) coordinates on the axis.

Compared to HCs, T2DM patients showed significant decreases in FC between the PCC and ACC, right superior frontal gyrus (SFG.R), right medial frontal gyrus, and right angular gyrus. In contrast, PCC exhibited increased FC to the right superior temporal gyrus (STG.R) and CAL (**Figure 4** and **Table 2**).

**Table 2:** Abnormal functional connectivity of the PCC in type 2 diabetes patients without cognitive and microvascular complications compared with healthy controls.

Brain region	BA	Peak MNI coordinates					Cluster size (voxels)	Peak *t* score
		*X*		*Y*		*Z*		
Decreased regions								
Anterior cingulate cortex	32	7		42		30	281	−3.28
R superior frontal gyrus	10	18		60		24	220	−4.36
R medial frontal gyrus	8	30		9		60	138	−4.01
R angular gyrus	22	52		−51		29	109	−3.13
Increased regions								
R superior temporal gyrus	22	62		−18		8	128	3.26
Calcarine fissure	17	13		−62		7	491	3.68

AlphaSim-corrected threshold of p < 0.05. Positive t-values: T2DM > control subjects; negative t-values: T2DM < control subjects.

PCC, posterior cingulate cortex; BA, Brodmann’s area; MNI, Montreal Neurological Institute; X, Y, Z, coordinates of the primary peak locations in the MNI space; L, left; R, right.

### 3.3 Correlation Analysis Results

**Figure 5:**
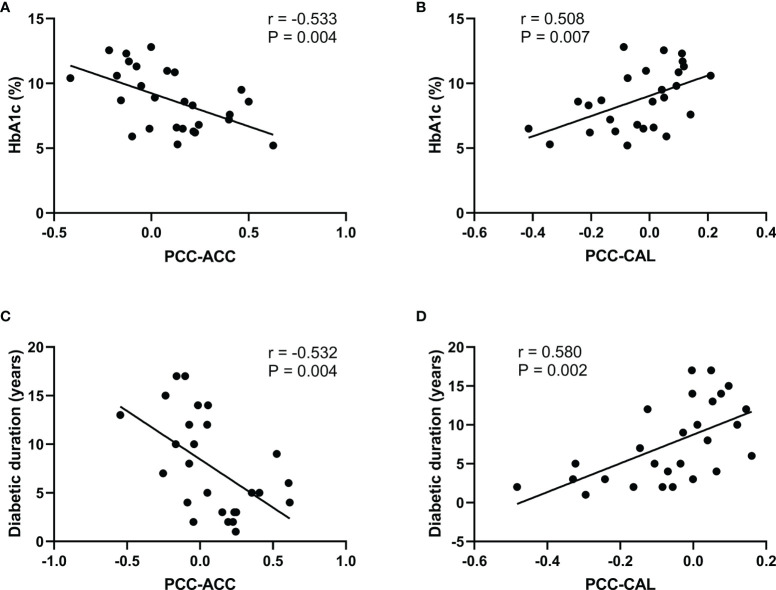
Significant correlations between the functional connectivity and different clinical variables in type 2 diabetes patients without cognitive and microvascular complications. **(A)** Correlation between the glycosylated hemoglobin (HbA1c) level and the functional connectivity of the posterior cingulate cortex and anterior cingulate cortex (PCC-ACC) (*r* = −0.533, *p* = 0.004). **(B)** Correlation between the HbA1c level and the functional connectivity of the PCC and calcarine fissure (CAL) (*r* = 0.508, *p* = 0.007). **(C)** Correlation between diabetes duration and the functional connectivity of PCC-ACC (*r* = −0.532, *p* = 0.004). **(D)** Correlation between diabetes duration and the functional connectivity of PCC-CAL (*r* = 0.580, *p* = 0.002).

**Figure 5** shows the significant correlation between the FC-PCC of ROI and the clinical indicators within the T2DM group. The FC strength between the PCC and ACC was only negatively correlated with HbA1c (*r* = −0.533, *p* = 0.004) and diabetes duration (*r* = −0.532, *p* = 0.004), while the FC strength between the PCC and CAL was only positively correlated with HbA1c (*r* = 0.508, *p* = 0.007) and diabetes duration (*r* = 0.580, *p* = 0.002). No significant correlation was found in healthy controls.

## 4 Discussion

In this study, T2DM patients without cognitive impairment and microvascular complications were included through strict screening criteria to avoid the impact of cognitive and microvascular diseases on FC. Both strengthened and weakened FCs of the PCC were found, and positive and negative correlations were also found between the FC-PCC and clinical indicators.

Functional connectivity and morphological studies have shown the aberrant brain structure and function in T2DM patients (3, 9). A decreased FC between the PCC and ACC, which are critical regions responsible for higher-order cognitive control (24), was observed. The aberrant FC, perfusion, and neural activity of ACC are often observed in MCI, which is assumed to represent a compensatory mechanism of neuropathology (25). What is more is that the FC of PCC-ACC was negatively correlated with HbA1c in our study. We speculate that hyperglycemia plays a key role in T2DM with progression into MCI. Recruitment of T2DM patients with MCI and comparisons of the FCs of PCC-ACC among T2DM patients without MCI and HCs were expected to determine whether this compensatory mechanism exists in T2DM, MCI, or both T2DM and MCI.

T2DM patients have an increased prevalence of depressive symptoms (26), and depression remains undiagnosed in as high as 50%–75% of diabetic patients (27). The abnormal STG and SFG were important biomarkers in depression patients (28). Previous studies (29, 30) showed that the amplitude of low-frequency fluctuation values of the STG and the regional homogeneity values of the SFG were significantly decreased in T2DM patients compared with HCs. Furthermore, in our study, reduced FC of PCC-SFG and increased FC of PCC-STG were found, even though MoCA and MMSE were used to exclude all cognitive disorder patients. The reasons may be that: a) the simple versions of MoCA and MMSE were used, which are sensitive to cognitive impairment, not psychiatric disorders; b) these patients may only be mildly depressed and difficult to identify. In future studies, the Depression Scale should be used and the effects of depression on the changes in brain structure and function in diabetic patients should be isolated.

Recently, one large sample of longitudinal study has shown that higher serum fasting insulin and insulin resistance could predict poorer verbal fluency and a steeper decline in verbal fluency (31). The middle temporal gyrus is linked to verbal fluency (32) and language processing (33). However, no significant alterations in the middle temporal gyrus were found in our study, which may be due to the patients we enrolled having no cognitive impairment. The aberrations in the structural or functional connections of the middle temporal gyrus reported in other literatures (12) may be due to the increased insulin resistance caused by long-term use of drugs in T2DM patients, which in turn affects language fluency.

Diabetic retinopathy is one of the most important complications of diabetes mellitus and is also one of the major causes of acquired blindness (34) in adults. As is known, more than 50% of T2DM patients would progress into having a decline in vision associated with retinopathy at the later stage (35). T2DM patients with concurrent retinopathy have structural and functional changes in the visual cortex (36). However, the neurological mechanism of this decline in vision remains unclear. It may be due to the central nervous system (visual cortex) dysfunction inhibiting the processing of peripheral afferent information, then reducing the need for peripheral retina, leading to retinopathy. It may also be due to the decrease of input information to the central nervous system after retinopathy occurrence results in a central nervous system disorder. Our study found that the strength of FC in the PCC-CAL increased and was positively correlated with HbA1c. Previous fMRI studies have indicated that decreased neural activity in the occipital area and CAL were related to visual impairment (37). The CAL is the part of the cerebral cortex that is the key node to receive and process visual information input from the lateral geniculate body (38). We speculate that hyperglycemia may enhance the activity of the visual cortex at the early stage and would deteriorate the activity of the visual cortex at the final stage. Strict glycemic control might delay and prevent the occurrence of diabetes-related complications besides visual impairment.

One of the most controversial procedures in the analysis of rs-fMRI data is global signal regression (GSR). Studies (39) have shown that the addition of GSR improved the performance of nearly all pipelines on most benchmarks, but exacerbated the distance dependence of the correlations between motion and FC. We compared the results with or without global mean signal regression in the preprocessing. The results showed that only three brain regions were found with significant differences without global mean signal regression, namely, the SFG, the middle frontal gyrus, and CAL, as shown in **Supplementary Figure S1**. The results with global mean signal regression found not only changes in the above three brain areas but also significant differences in the ACC, angular gyrus, and STG, as shown in **Figure 4**. As for the cause of this result, further research is needed.

Our study has several limitations. Firstly, the study is cross-sectional with a relatively small sample size. In a next step, the sample number should be increased and longitudinal tracking should be carried out to further establish the causal relationship between hyperglycemia and FC. Secondly, the lack of assessment of the insulin levels and depression in this study somewhat limited our conclusions.

Despite these limitations, the findings have important clinical implications. The FC, especially of the PCC with cognitive and visual brain regions, was altered before clinically measurable cognitive impairment and microvascular complications occurred in T2DM patients. In addition, the FC of the PCC with cognitive and visual brain regions was correlated with HbA1c and diabetes duration. This indicates that clinicians should pay attention not only to blood glucose control but also to brain function changes before the occurrence of adverse complications, which is of great significance for the prevention of cognitive dysfunction and visual impairment.

## Data Availability Statement

The raw data supporting the conclusions of this article will be made available by the authors, without undue reservation.

## Ethics Statement

The studies involving human participants were reviewed and approved by the Research Ethics Committee of the Affiliated Liyuan Hospital, Tongji Medical College, Huazhong University of Science and Technology. The patients/participants provided written informed consent to participate in this study.

## Author Contributions

Study concept and design, acquisition of data, analysis and interpretation of data, drafting of the manuscript, critical revision of the manuscript for important intellectual content, material support and study supervision: All authors. All authors have seen and agree with the content of the last version of manuscript.

## Funding

This work was supported in part by the Ministry of Science and Technology of China (2015CB351701), the Fundamental Research Funds for the Central Universities (Huazhong University of Science and Technology, no. 2013QN129), the National Natural Science Foundation of China (31730039, 31671133), National Major Scientific Instruments and Equipment Development Project (ZDYZ2015-2), and the Chinese Academy of Sciences Strategic Priority Research Program B grants (XDB32010300).

## Conflict of Interest

The authors declare that the research was conducted in the absence of any commercial or financial relationships that could be construed as a potential conflict of interest.

## Publisher’s Note

All claims expressed in this article are solely those of the authors and do not necessarily represent those of their affiliated organizations, or those of the publisher, the editors and the reviewers. Any product that may be evaluated in this article, or claim that may be made by its manufacturer, is not guaranteed or endorsed by the publisher.
