# Supplementary figures and images for: Aberrant Functional Connectivity of the Posterior Cingulate Cortex in Type 2 Diabetes Without Cognitive Impairment and Microvascular Complications
Source: Front Endocrinol (Lausanne). 2021 Oct 25;12:722861. doi: 10.3389/fendo.2021.722861 (PMC8573207; doi:10.3389/fendo.2021.722861)

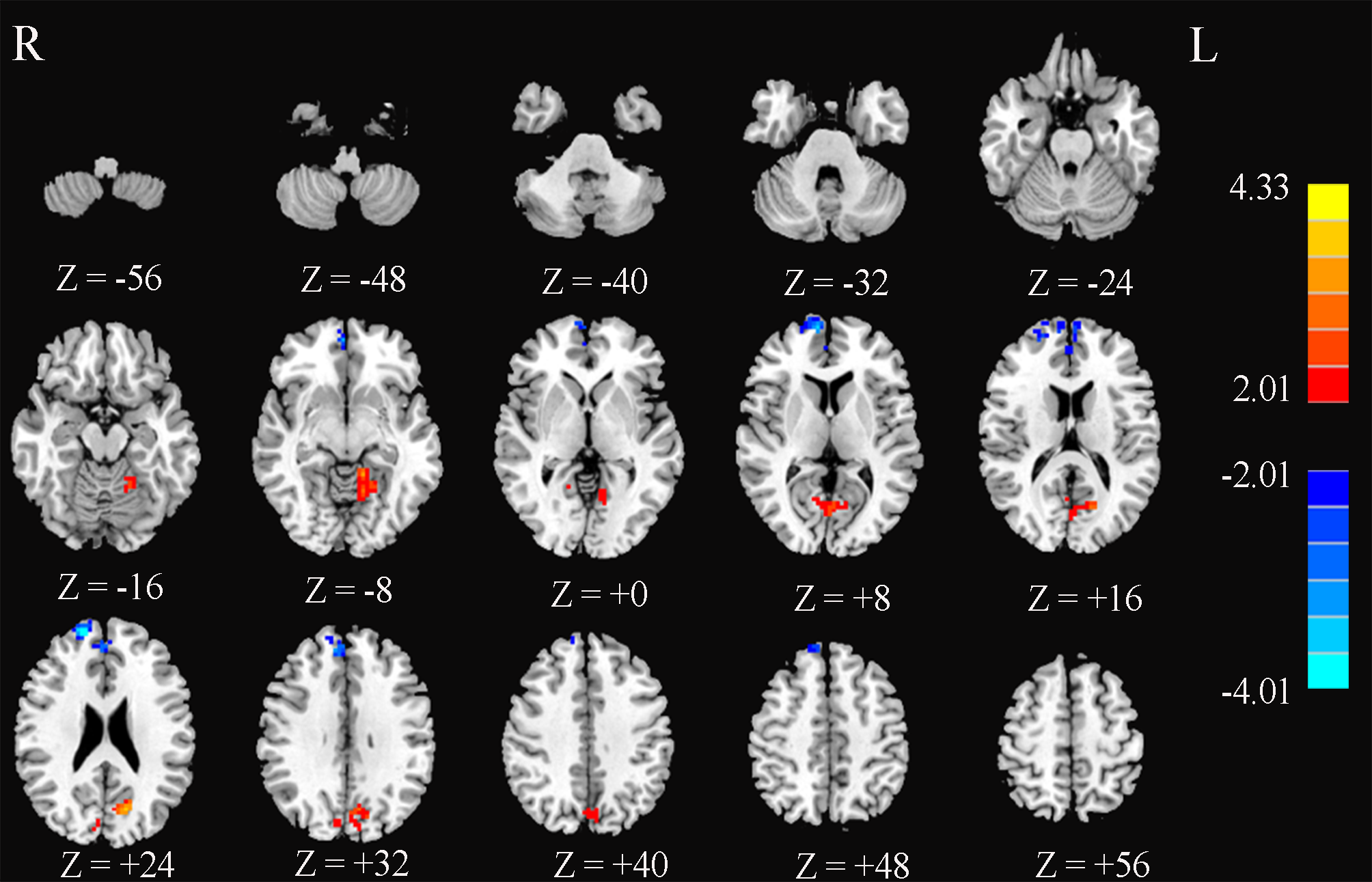

Supplement: Supplementary file 1 [file Image_1.tif]
